# Identification of Cysteine Protease Inhibitor CST2 as a Potential Biomarker for Colorectal Cancer

**DOI:** 10.7150/jca.53983

**Published:** 2021-06-22

**Authors:** Qiurong Xie, Liya Liu, Xiaoping Chen, Ying Cheng, Jiapeng Li, Xiuli Zhang, Nanhui Xu, Yuying Han, Huixin Liu, Lihui Wei, Jun Peng, Aling Shen

**Affiliations:** 1 Academy of Integrative Medicine, Fujian University of Traditional Chinese Medicine, 1 Qiuyang Road, Minhou Shangjie, Fuzhou, Fujian 350122, China.; 2 Fujian Key Laboratory of Integrative Medicine on Geriatrics, Fujian University of Traditional Chinese Medicine, 1 Qiuyang Road, Minhou Shangjie, Fuzhou, Fujian 350122, China.; 3 Department of Physical Education, Fujian University of Traditional Chinese Medicine, 1 Qiuyang Road, Minhou Shangjie, Fuzhou, Fujian 350122, China.

**Keywords:** Colorectal cancer, CST2, Tissue microarray, Oncogene, Biomarker

## Abstract

Additional biomarkers for the development and progression of colorectal cancer (CRC) remain to be identified. Hence, the current study aimed to identify potential diagnostic markers for CRC. Analyses of cysteine protease inhibitor [cystatins (CSTs)] expression in CRC samples and its correlation with cancer stage or survival in patients with CRC demonstrated that CRC tissues had greater CST1 and CST2 mRNA expression compared to noncancerous adjacent tissues, while higher CST2 mRNA expression in CRC tissues was correlated with advanced stages and disease-free survival in patients with CRC, encouraging further exploration on the role of CST2 in CRC. Through an online database search and tissue microarray (TMA), we confirmed that CRC samples had higher CST2 expression compared to noncancerous adjacent tissue or normal colorectal tissues at both the mRNA and protein levels. TMA also revealed that colorectal adenoma, CRC, and metastatic CRC tissues exhibited a significantly increased CST2 protein expression. Accordingly, survival analysis demonstrated that the increase in CST2 protein expression was correlated with shorter overall survival of patients with CRC. Moreover, our results found a significant upregulation of CST2 in multiple cancer tissues. Taken together, these findings suggest the potential role of CST2 as a diagnostic and prognostic biomarker for CRC.

## Introduction

Colorectal cancer (CRC) is the third most common malignancy and the fourth leading cause of cancer-related deaths worldwide 1, with more than 500,000 individuals dying of CRC annually 2-5. Despite extensive research into the mechanisms of CRC development and progression, the etiology of CRC remains largely unclear. Although patients with local colon cancer generally undergo resection as the first-line treatment, colon cancer recurrence and metastasis still occur after surgery, with distant metastasis being the leading cause of treatment failure and death in such patients. Given the high morbidity and mortality of CRC, identifying more valuable and convenient biomarkers for early diagnosis, prevention, and personalized therapy is considerably important and urgent.

Bioinformatics and omics provide powerful tools for the study of complex diseases, including tumors 6-10. Microarray-based gene expression assessment has been the most commonly used and successful high-throughput technique utilized to study complicated disease pathogenesis 11. To determine which genes are involved in CRC, this study also used the Colon adenocarcinoma (COAD) and Rectum adenocarcinoma (READ) datasets from The Cancer Genome Atlas (TCGA) for gene expression analysis. Among the differentially expressed genes (DEGs), especially within the CST family, we had found significant upregulation of CST2 mRNA expression in CRC tissues, with high CST2 expression being closely associated with shorter overall survival (OS) and disease-free survival (DFS) in patients with CRC, suggesting that the CST2 gene may play an important role in tumor development.

Cysteine protease inhibitors [cystatins (CSTs)], named for its inhibitory effect on cysteine protease, were originally a class of proteins first discovered in egg white by Anastasi using affinity chromatography that had been found to play a crucial role in maintaining cellular protein balance 12, 13. Cysteine protease inhibitors can be divided into four categories based on its molecular structure: (1) the stefins family: mainly intracellular proteins consisting of approximately 100 amino acid residues and comprising mainly stefin A, stefin B, etc.; (2) the cystatins family (type 2 cystatins): secreted proteins consisting of approximately 120 amino acids, including Cystatin SN (CST1), Cystatin SA (CST2), Cystatin C (CST3), Cystatin S (CST4), and Cystatin D (CST5); (3) the kininogens family (type 3 cystatins): glycosylated proteins distributed across blood vessels, plasma, synovial fluid, and amniotic fluid and consisting of 3 discrete classes termed H-Kininogens, L-Kininogens, and T-Kininogens 14; and (4) the phytocystatins family: including all plant cysteine proteinase inhibitors 15. Among the aforementioned cysteine protease inhibitors, the human type 2 cystatins are encoded by a multigene family of eight to nine members, with all the loci mapped to 20p11.2. 16, 17. The members of this family share a common cystatin motif, which is the active area of cystatins 18. A balance in the levels of cysteine proteases and CSTs needs to be maintained in the body, with a disturbance in such a balance leading to the occurrence, development, invasion, and metastasis of tumors 19, 20.

Previous studies have shown that CST1 overexpression is closely associated with the proliferation, invasion, and metastasis of malignant tumors, such as colorectal, gastric, pancreatic, and other cancers 21-24. Cystatin SA (CST2) encodes a secreted thiol protease inhibitor found at high levels in the saliva, tears, and seminal plasma. Results from the SELDI Protein Chip platform revealed that cystatin proteins SA-I may be a useful tumor biomarker for oral squamous cell carcinoma 25. CST3 encodes the most abundant extracellular inhibitor of cysteine proteases, which is virtually expressed in all body organs, albeit at higher amounts in biological fluids. Reports have shown that hepatocellular carcinoma tissues exhibit significantly higher levels of CST3, CSTA, and CSTB compared to adjacent healthy tissue 26, 27, with CST3 possibly functioning as a serum marker for hepatocellular carcinoma 28. However, patients with bladder cancer (BCa) exhibited lower serum CST3 concentrations than the control group, making it a potential marker for BCa but not its aggressiveness 29. CST3 also plays a protective role against inflammation and cancer pathogenesis by inhibiting cathepsins 30. CST4, on the other hand, encodes a type 2 salivary cysteine peptidase inhibitor (a S-type cystatin) that has high expression levels in the saliva, tears, and seminal plasma. Consistent with upregulated levels in gastrointestinal cancer tissues and cell lines 31, *in vivo* results revealed that CST4 overexpression significantly promoted gastric cancer cell tumorigenicity 32. Cystatin D/CST5 encodes an endogenous inhibitor of cysteine proteases in the cathepsin family and is directly induced by the vitamin D receptor (VDR) 33. In CRC cells, p53-induced upregulation of CST5 promoted the induction of mesenchymal-epithelial transition 34. Recently, CST5 had been shown to inhibit the proliferation, migration, and tumor formation of xenografted CRC cell lines 35, as well as exhibit higher expression levels in hepatocellular carcinoma tissue compared to normal tissue 36. Thus, CSTs cannot be merely regarded as a cysteine protease inhibitor considering its vital role in tumorigenesis.

The current study analyzed CST expression in CRC and elucidated the relationship between CST expression and patient survival determined using bioinformatics analysis and immunohistochemical (IHC) experiments. Our results suggested that high CST2 expression in CRC was closely associated with patient prognosis, making it a potential diagnostic biomarker for CRC.

## Materials and methods

### Bioinformatics analysis

The current study compared CST mRNA expression between CRC and normal tissues and determined the correlation between its expression and OS or DFS among patients with CRC using GEPIA (http://gepia.cancer-pku.cn/) and Oncomine (http://www.oncomine.org). CST mRNA expression across different CRC stages were analyzed using GEPIA (http://gepia.cancer-pku.cn/). CST2 expression level and prognosis across different cancers were analyzed using TIMER (https://cistrome.shinyapps.io/timer/). Patients with CRC were classified into low and high groups based on the expression of CSTs and then analyzed using the log-rank test.

### Immunohistochemistry (IHC)-based tissue microarray and survival analysis

Tissue microarray chips for CRC samples were obtained from Shanghai Outdo Biotech Company (Shanghai, China; Cat#: HColA180Su15; HColAde080CD01). The patients who have not received chemotherapy or radiotherapy and with no other diseases were selected. Clinic pathological features of CRC patients were summarized in Table 2 and Supplement Table S1. IHC analysis was conducted using an antibody against CST2 (Rabbit polyclonal to CST2; dilution 1:500; SAB, Cat#34629; USA). Scoring was conducted by two experienced pathologists blinded to tissue identity using a grading system based on staining intensity (no staining, 0; weak, 1; moderate, 2; strong, 3) and percentage of positively stained cells (1%-25% positive, 1; 26%-50%, 2; 51%-75%, 3; 76%-100%, 4). The final score was calculated as an intensity score × percentage score. Kaplan-Meier survival curves were plotted for low- (IHC score ≤ 3) and high-expression (IHC score > 3) groups and then analyzed using the log-rank test.

### Statistical analysis

Statistical analyses were performed using SPSS 20.0 software. Our results evaluated using the Wilcoxon test and expressed as means ± standard deviation from all independent samples. Survival data were analyzed using the Kaplan-Meier method and compared using the log-rank test. *P* values less than 0.05 indicated statistical significance.

## Results

### Cystatin mRNA expression in colorectal cancer tissues

**Figure 1:**
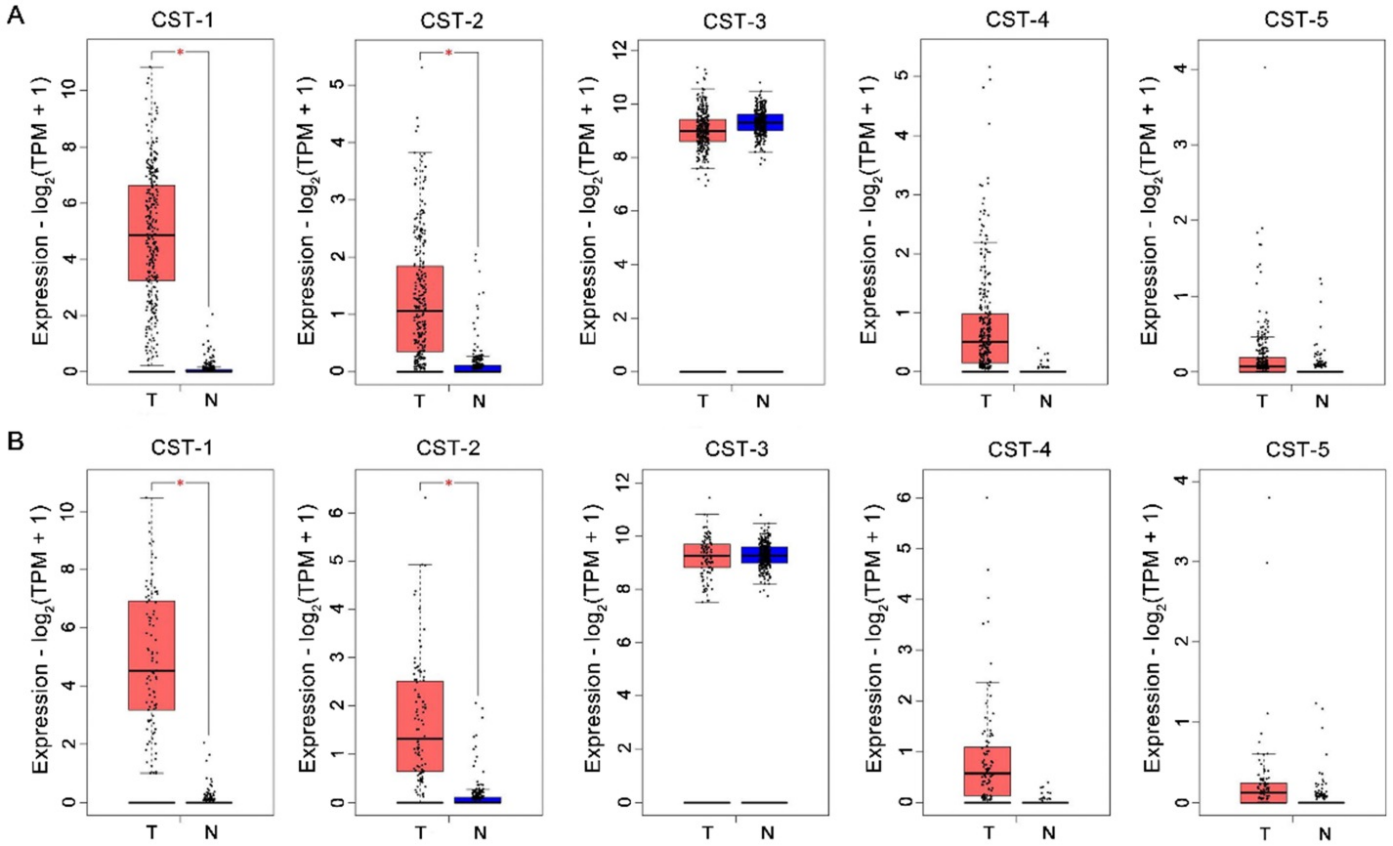
** Upregulation of CST1 and CST2 at the mRNA level in colorectal cancer tissues.** CST mRNA expression was analyzed using the (A) Colon adenocarcinoma (COAD) and (B) Rectum adenocarcinoma (READ) datasets from the GEPIA database. *P < 0.05, tumor vs. normal tissues. T, tumor tissues; N, normal tissues.

**Figure 2:**
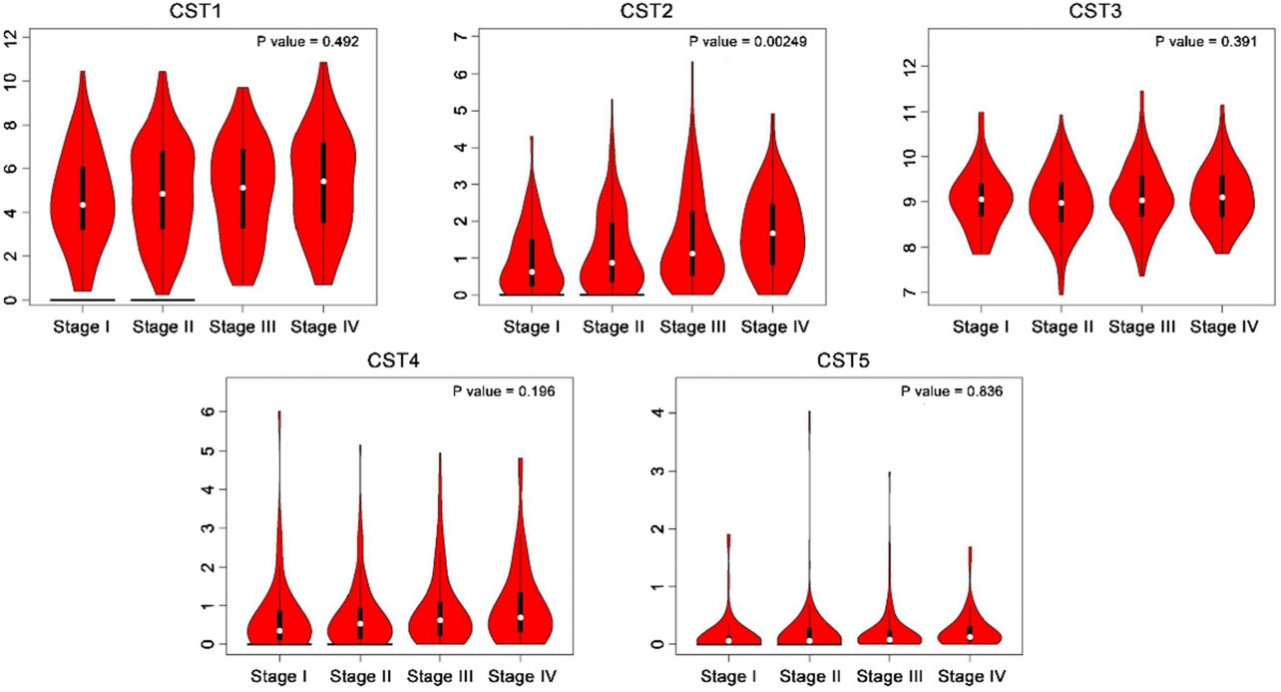
** Higher expression of CST2 in advanced stage colorectal cancer.** CST mRNA expression at different colorectal cancer stages was compared based on the GEPIA database.

CST mRNA expression in CRC and normal tissues was compared based on the GEPIA database. As shown in Fig. 1, mRNA levels of both CST1 and CST2 were significantly higher in CRC tissues than in normal tissues, although no significant difference in other mRNA expressions were noted between CRC and normal tissues. After analyzing CST expression across different CRC stages using the GEPIA database, our results showed that advanced CRC tissues had significant CST2 upregulation, whereas no other CST showed a significant difference according to CRC stage (see Fig. 2).

### Correlation between cystatin expression and survival

**Figure 3:**
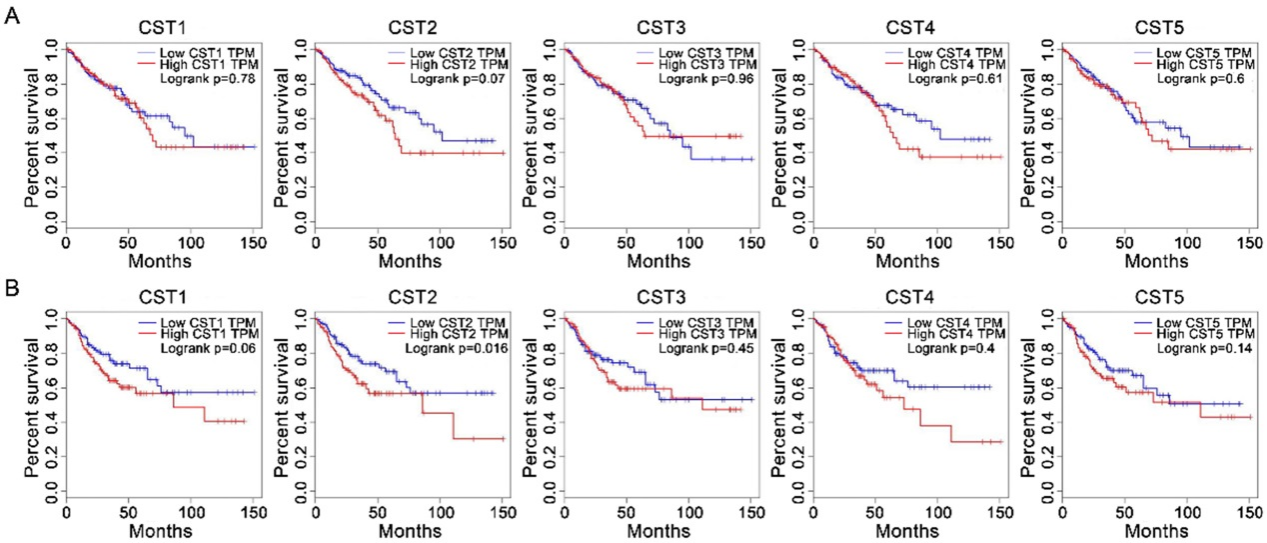
** High CST2 mRNA expression was correlated with shorter disease-free survival in patients with colorectal cancer.** The correlation between overall survival (A) and disease-free survival (B) in patients with CRC and CST mRNA expression in CRC tissues was analyzed through the GEPIA database. Survival was analyzed using the log-rank test.

After analyzing the association between CST and OS or DFS in patients with CRC using the Kaplan-Meier method through the GEPIA website, our results showed that high CST2 expression in CRC tissues was significantly correlated with DFS (*P* < 0.05, Fig. 3) but not with OS (log-rank *P* = 0.07). However, no significant difference between low and high groups were observed for other CST proteins.

### High CST2 expression in colorectal adenoma and carcinoma tissues

**Figure 4:**
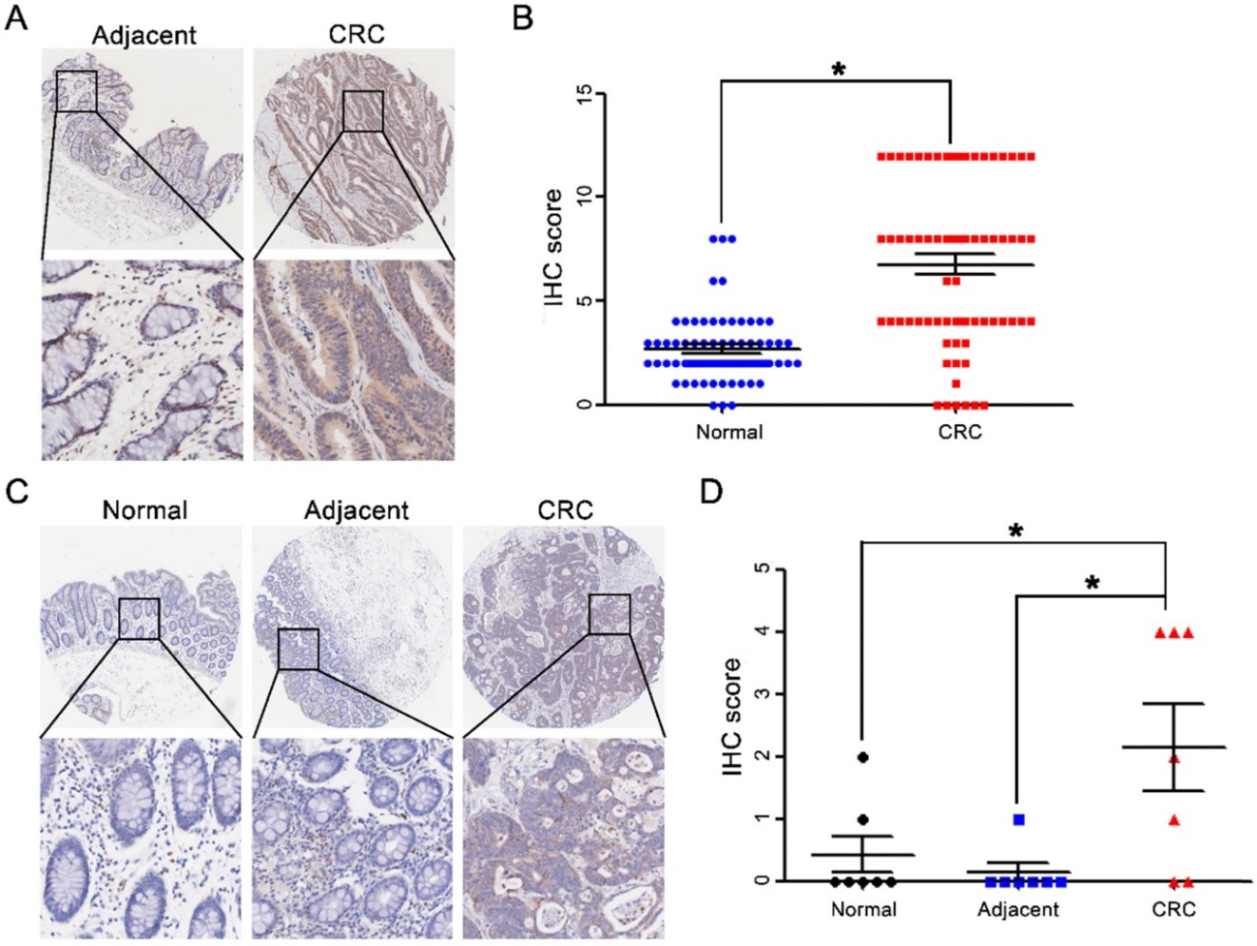
** CST2 upregulation at the protein level in colorectal cancer tissues.** Microscopic observation (A) and scatter chart (B) of CST2 protein in 71 pairs of colorectal cancer tissues and noncancerous adjacent tissues, which was determined by immunohistochemistry-based tissue microarray. Image magnification at ×40 or ×200. *P < 0.05, tumor vs. normal tissues. Microscopic observation (C) and scatter chart (D) of CST2 protein in 7 pairs of CRC tissues, normal, and adjacent tissues determined using immunohistochemistry-based tissue microarray. Image magnification at ×40 or ×200. *P < 0.05, tumor vs. normal tissues, tumor vs. adjacent.

**Figure 5:**
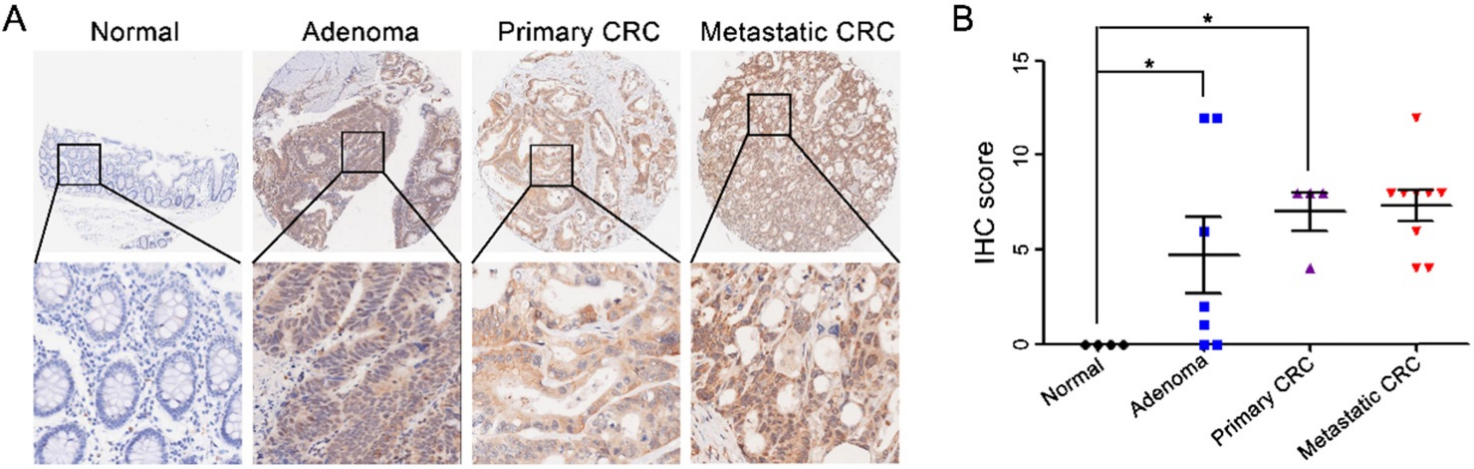
** CST2 upregulation at the protein level in both colorectal adenoma and carcinoma tissues.** Microscopic observation (A) and scatter chart (B) of CST2 protein in normal, adenoma, primary and metastatic colorectal tissues determined using immunohistochemistry-based tissue microarray. Image magnification at ×40 or ×200. *P < 0.05, colorectal cancer vs. normal tissues.

After comparing CST2 expression between CRC and normal tissues based on the Oncomine database, our results showed that CST2 mRNA levels in CRC tissues were approximately 2-6-fold higher relative to normal tissues (Table 1). The upregulation of CST2 was verified by IHC-based TMA of 71 pairs of CRC and adjacent noncancerous tissues (Fig. 4A and 4B) and 7 pairs of CRC tissues and adjacent noncancerous tissue, as well as normal tissues (Fig. 4C and 4D). Furthermore, after comparing CST2 expression in colorectal adenoma, carcinoma, and metastatic tissues (Fig. 5), our results showed that CST2 was significantly upregulated in colorectal adenoma and CRC tissues, although no significant difference between primary and metastatic CRC tissues were observed.

### Correlation between CST2 and shorter overall survival

**Figure 6:**
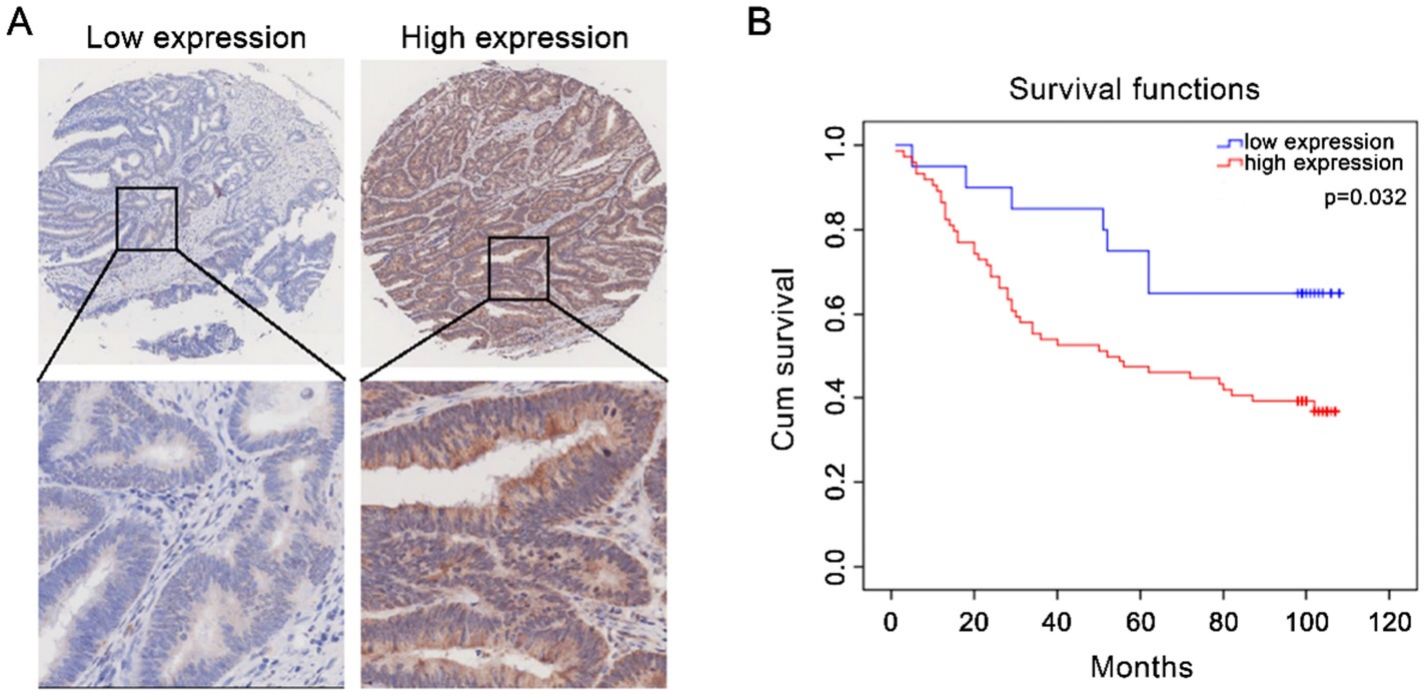
** High CST2 protein expression was correlated with shorter overall survival in patients with colorectal cancer.** (A) Representative image of high- and low-CST2 expression. Magnification at ×40 or ×200. (B) Correlation between CST2 protein (immunohistochemistry-based tissue microarray) expression and overall survival was analyzed using Kaplan-Meier plots in 94 patients with colorectal cancer (*P* < 0.05).

The association between CST expression and survival in patients with CRC was further analyzed based on the protein expression of CST2 in 94 CRC tissues. As shown in Fig. 6, higher CST2 expression was significantly correlated with shorter OS in patients with CRC (*P* < 0.05), suggesting the potential for CST2 as a prognostic marker for CRC. Consistently, analysis of the clinicopathological characteristics in 94 patients with CRC showed higher OS rates with lower CST2 expression (Table 2) detected using IHC-based TMA.

### High CST2 expression in multiple cancer tissues

**Figure 7:**
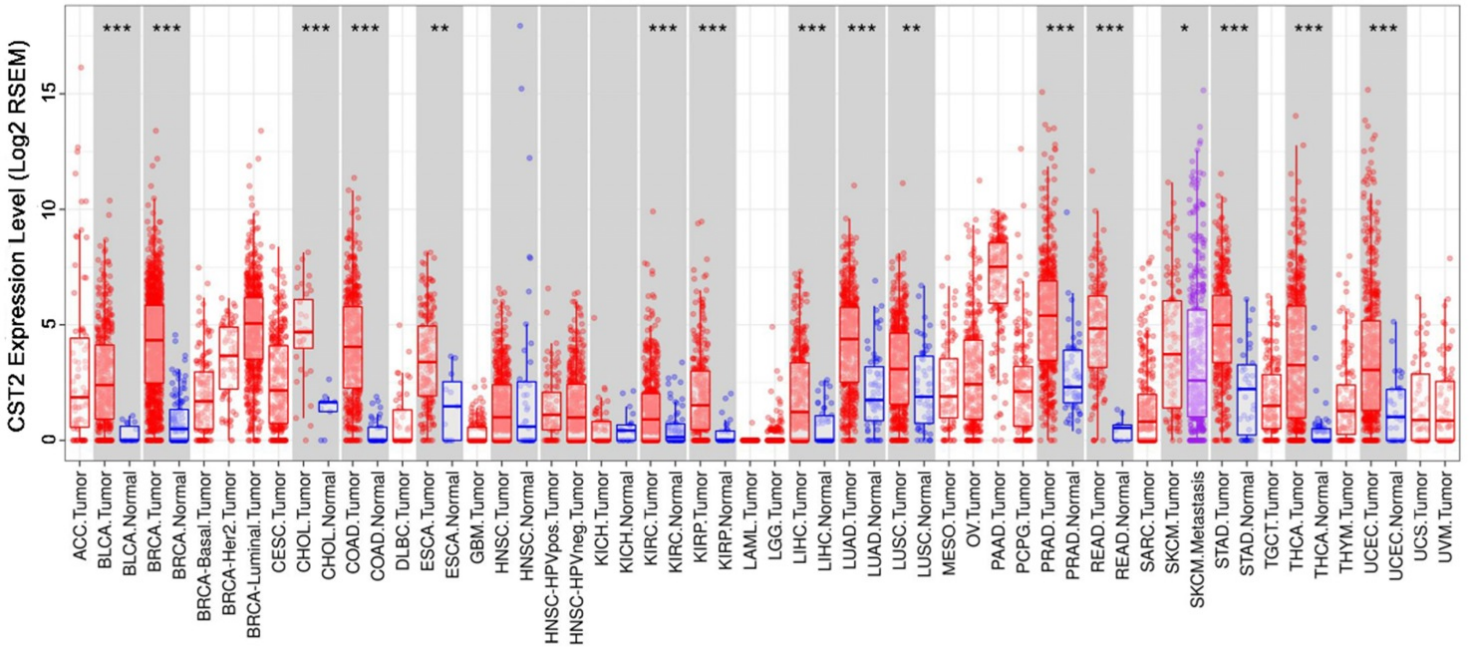
** CST2 was highly expressed in multiple cancer tissues.** CST2 expression in multiple cancer tissues and normal tissues was compared based on the STAD database. *P < 0.05, **P < 0.005, ***P < 0.001, tumor vs. normal tissues.

After comparing CST2 expression in multiple cancer and normal tissues based on the TIMER database, our results showed that several cancer tissues, including bladder carcinoma, breast cancer, cholangiocarcinoma, COAD, esophageal carcinoma, kidney renal clear cell carcinoma, kidney renal papillary cell carcinoma, liver hepatocellular, lung adenocarcinoma, lung squamous cell carcinoma, prostate adenocarcinoma, pulmonary enteric adenocarcinoma, stomach adenocarcinoma, thyroid cancer, uterine corpus endometrial carcinoma, and others, had higher CST2 mRNA levels relative to normal tissues (Fig. 7). This suggests that CST2 upregulation might be a common occurrence and play an essential role during the development of various types of cancers.

## Discussion

CRC, the third most common malignancy and fourth leading cause of cancer-related death worldwide, requires more extensive research to elucidate its underlying mechanism 1-4. Considering the possible occurrence of metastasis, drug resistance, and tumor recurrence after initial treatment, identifying additional biomarkers for early diagnosis, prevention, and personalized therapy is critical and urgent. Interestingly, the current study found a definite upregulation of both CST1 and CST2 in CRC tissues, while higher CST2 expression in CRC tissues was correlated with advanced clinical stages and shorter DFS in patients with CRC. Consistently, database analysis and IHC-based TMA analysis confirmed that colorectal adenoma and carcinoma tissues had clearly higher CST2 expression at both the mRNA and/or protein level compared to normal colorectal tissues or matched noncancerous adjacent tissues. Moreover, higher CST2 expression at the protein level had been found to be significantly associated with shorter OS in patients with CRC. The current study also showed that CST2 mRNA expression was upregulated in multiple types of cancers. The aforementioned findings suggest the potential role of CST2 as a marker for early diagnosis and prognosis.

CST2 is a member of the cysteine protease inhibitors, the genes of which encode the S-type (salivary) cystatin SA 37, 38. Previous studies have revealed that CST2 might play a positive role in predicting the course of some diseases, such as allergic rhinitis 39, chronic rhinosinusitis with nasal polyps 40, and periodontal disease 41. The present study found that CRC tissues had higher CST1 and CST2 mRNA expression compared to normal tissues based on two different online databases, (i.e., GEPIA and Oncomine). Consistently, IHC-based TMA analysis further confirmed that colorectal adenoma and carcinoma tissues had clearly higher CST2 protein expression compared to normal colorectal tissues, suggesting the potential involvement of CST2 in the development of CRC and its possibly utility as an early diagnostic marker. However, more clinical samples are needed to further verify the early diagnostic value of CST2 for CRC. Consistent with a previous study 21, the current study also found significant upregulation of CST1 in CRC tissues, although not correlation had been observed between CST1 expression and clinical stage or survival of patients with CRC. Although previous studies have demonstrated that CST3 was upregulated in both hepatocellular carcinoma tissues and serum, suggesting its potential as a marker for hepatocellular carcinoma 26-28, the current study found no significant difference in CST3, CST4, and CST5 expression between CRC tissues and noncancerous CRC tissues, as well as no correlation between CST3, CST4, and CST5 and advanced stage or OS in patients with CRC. These findings encouraged further exploration on the potential oncogenic activity of CST2 in CRC.

Further analysis of the correlation between CST2 expression and survival in patients with CRC revealed that higher CST2 expression was significantly correlated with advanced CRC stages, while no difference in CST1, CST3, CST4, and CST5 expression had been noted according to CRC stage. Unfortunately, our results showed no significant difference in CST2 protein expression between primary and metastatic CRC tissues. Moreover, database analyses and IHC-based TMA revealed a significantly association between higher CST2 expression and shorter survival in patients with CRC. These results suggest the involvement of increased CST2 expression in CRC progression and its potential as a prognostic prediction marker, which needs to be further verified using larger sample sizes and diverse ethnic backgrounds.

In conclusion, the present study revealed that colorectal adenoma and carcinoma tissues had significantly higher CST2 expression compared to noncancerous colorectal tissues at both the mRNA and protein levels. Moreover, higher CST2 expression in CRC was significantly correlated with shorter survival and advanced stage in patients with CRC. However, further studies are needed to explore the biological function of CST2 in CRC.

## Supplementary Material

Supplementary table.

## Figures and Tables

**Table 1:** CST2 mRNA expression in colorectal cancer tissues.

Dataset		Fold Change	P value	t-test
TCGA Colorectal	Colon Adenocarcinoma vs. Normal	5.610	1.29E-23	14.651
TCGA Colorectal	Rectal Adenocarcinoma vs. Normal	5.520	3.72E-21	13.071
TCGA Colorectal	Rectal Mucinous Adenocarcinoma vs. Normal	2.989	1.07E-6	9.288
TCGA Colorectal	Cecum Adenocarcinoma vs. Normal	4.335	1.24E-10	8.893
TCGA Colorectal	Colon Mucinous Adenocarcinoma vs. Normal	3.175	2.85E-7	6.449
TCGA Colorectal	Rectosigmoid Adenocarcinoma vs. Normal	4.107	0.013	4.977
Skrzypczak Colorectal	Colorectal Carcinoma vs. Normal	1.996	2.24E-10	7.874
Kaiser Colon	Cecum Adenocarcinoma vs. Normal	1.589	1.68E-5	5.445
Kaiser Colon	Rectal Mucinous Adenocarcinoma vs. Normal	1.771	2.20E-2	3.102
Gaedcke Colorectal	Rectal Adenocarcinoma vs. Normal	1.947	1.50E-13	9.05
Hong Colorectal	Colorectal Carcinoma vs. Normal	4.866	2.66E-5	7.332
Sabates-Bellver Colon	Colon Adenoma vs.Normal	2.615	5.21E-6	4.922
Sabates-Bellver Colon	Rectal Adenoma vs. Normal	1.951	2.80E-2	2.206

CST2 expression in colorectal cancer tissues and normal tissues was compared based on the Oncomine database.

**Table 2:** Association between CST2 expression and clinicopathological characteristics in patients with colon cancer.

Characteristic	n	CST2	CST2	Chi-Square	P value
		Low expression	High expression		
**Total**	94	20	74		
**Gender**					
Male	51	13	38	7.084	0.008**
Female	42	7	35		
**Age**					
< 65	35	11	24	0.039	0.843
≥ 65	58	9	49		
**Clinical stage**					
Ⅰ + Ⅱ	49	11	38	0.062	0.803
Ⅲ + Ⅳ	45	9	36		
**T stage**					
T1 + T2	6	1	5	1.340	0.213
T3 + T4	84	19	65		
**N stage**					
N0	56	13	43	2.613	0.125
N1 + N2	37	7	30		
**M stage**					
M0	91	20	71	0.785	0.375
M1	3	0	3		
**Patient status**					
live	41	13	28	11.545	0.001**
dead	53	7	46		
**Tumor size**					
< 5 cm	37	8	29	0.147	0.701
≥ 5 cm	55	12	43		

## Abbreviations

CRC: colorectal cancer
CST: cystatin
DFS: disease-free survival
TMA: tissue microarray
OS: overall survival
COAD: colon adenocarcinoma
READ: rectum adenocarcinoma
TCGA: the cancer genome atlas
DEGs: differential expressed genes
BCa: bladder cancer
VDR: vitamin D receptor
IHC: immunohistochemical
