# Supplementary material for: Identification of Cysteine Protease Inhibitor CST2 as a Potential Biomarker for Colorectal Cancer
Source: J Cancer. 2021 Jun 22;12(17):5144–52. doi: 10.7150/jca.53983 (PMC8317524; doi:10.7150/jca.53983)
Supplement: Supplementary file 1 — Supplementary table. [file jcav12p5144s1.pdf]

Supplementary Table S1. Clinic pathological features of 14 CRC patients

Characteristic		n (%)
Age	< 65	9 (64)
	≥ 65	5 (36)
Gender	Male	8 (57)
	Female	6 (43)
Tumor location	Colon	14 (100)
	Rectum	0 (0)
Clinical stage	I+ II	11 (79)
	III + IV	3 (21)
T stage	T1+T2	6 (43)
	T3+T4	8 (57)
N stage	N0	5 (36)
	N1	6 (43)
	N2	3 (21)
M stage	M0	9 (64)
	M1	5 (36)
Distant metastasis		5 (36)
